# CEP20 promotes invasion and metastasis of non-small cell lung cancer cells by depolymerizing microtubules

**DOI:** 10.1038/s41598-023-44754-8

**Published:** 2023-10-14

**Authors:** Sijie Feng, Shuai Yuan, Baohua Hou, Zhiqiang Liu, Yanjun Xu, Shuangying Hao, Yunkun Lu

**Affiliations:** 1 School of Medicine, Henan Polytechnic University, Jiaozuo, China; 2 School of Medicine, Sir Run Run Shaw Hospital, Zhejiang University, Hangzhou, China; 3 Jiaozuo Key Laboratory of Gynecological Oncology Medicine, Jiaozuo, China; 4 Department of Medical Thoracic Oncology, The Cancer Hospital of the University of Chinese Academy of Sciences (Zhejiang Cancer Hospital), Institute of Basic Medicine and Cancer (IBMC), Chinese Academy of Sciences, Hangzhou, China

**Keywords:** Non-small-cell lung cancer, Cell migration, Cytoskeleton, Cancer, Cell biology, Biomarkers

## Abstract

Worldwide, Lung cancer is the leading cause of cancer-related death and poses a direct health threat, non-small cell lung cancer (NSCLC) is the most common type. In this study, we demonstrated that centrosomal protein 20 (CEP20) is upregulated in NSCLC tissues and associated with cancer invasion metastasis. Notably, CEP20 depletion inhibited NSCLC cell proliferation, migration, and microtubule polymerization. Mechanistically, we discovered that CEP20 is critical in the development of NSCLC by regulating microtubule dynamics and cell adhesion-related signaling pathways. Furthermore, the knockdown or overexpression of CEP20 affects microtubule polymerization in A549 cell lines. Our research provides a promising therapeutic target for the diagnosis and treatment of lung cancer, as well as a theoretical and experimental basis for clinical application.

## Introduction

Lung cancer is a highly prevalent malignant tumor worldwide and is associated with high mortality^1–3^. It is the leading cause of cancer-related death worldwide^1^. Lung cancer can be classified into two types based on the differentiation degrees and cell morphological features: small cell lung cancers (SCLCs) and non-small cell lung cancers (NSCLCs), with the latter being more common (~ 85%), and posing a greater threat to patients’ survival^4^. As NSCLCs are often asymptomatic in the early stages, most patients are diagnosed with mid to late-stage tumors for which resection is no longer feasible^4,5^. Furthermore, advanced NSCLCs exhibit robust invasion and migration, leading to poor prognoses despite comprehensive treatments, such as radiotherapy and adjuvant chemotherapy with multi-drug combinations^6^. Due to recent developments in diagnosis and pharmaceuticals, molecular targeted therapy, such as those targeting epidermal growth factor receptor (EGFR) and anaplastic lymphoma kinase (ALK), have become increasingly important in the treatment of NSCLCs, leading to improved prognoses of patients^7^. However, with prolonged medication, a large number of patients experience primary and secondary resistance and recurrence, necessitating the development of new drugs targeting promising molecules with clinical viability^6,7^. Thus, identifying new drug targets and developing novel anti-cancer drugs remains a priority in lung cancer research.

Microtubules are polarized cytoskeletal structures^8^, assembled from α-/β-tubulin heterodimers to form highly dynamic filaments, which play a crucial role in many physiological and pathological processes, including cell cycle and mobility^9,10^. Anti-tumor drugs targeting microtubules have recently been cumulatively approved against NSCLCs in clinical practice^11^. These drugs promote microtubule depolymerization or polymerization by utilizing their dynamic properties, ultimately altering the normal cellular processes. As a result, tumor cells are arrested in the mitotic cycle and undergo apoptosis and death. One of the most effective drugs is Paclitaxel, which binds to polymerized microtubules and promotes their assembly while inhibiting their depolymerization, thus forming stable non-functional microtubules^12^. Therefore, Paclitaxel acts as a tumor suppressor by reducing cell mitosis in various cancers, including ovarian, breast, and lung^12–14^. Aberrant expression of microtubule-related proteins, such as Stathmin, Tau, and protein family possessing Ras-associated domains, is well-reported in tumor cells^15–17^. These proteins affect cell mitosis and motility by inducing imbalances in microtubule dynamics, thereby playing significant roles in tumorigenesis and progression.

Centrosomal Protein 20 (CEP20), also known as FOP-Related Protein of 20 KDa (FOR20), localizes to both centrioles and centriolar satellites^18,19^. In mammalian cells, CEP20 is identified to recruit polo-like kinase 1 (PLK1) to centrosomes^20^ and participates in microtubule depolymerization^21^ and ciliogenesis^22^ by our previous works. CEP20 also plays an essential role during mammalian embryonic development, and its absence leads to impaired left–right patterning of embryos and disrupts angiogenesis in yolk sacs and embryos^23^. In this study, we revealed that CEP20 promotes malignant behavior in lung cancer cells by affecting microtubule assembly, highlighting its potential as a new promising drug target for the treatment of lung cancer. Together, our findings provide new insights into targeted therapy for lung cancer.

## Results

### *CEP20* is highly expressed in human non-small cell lung cancer tissues

**Figure 1:**
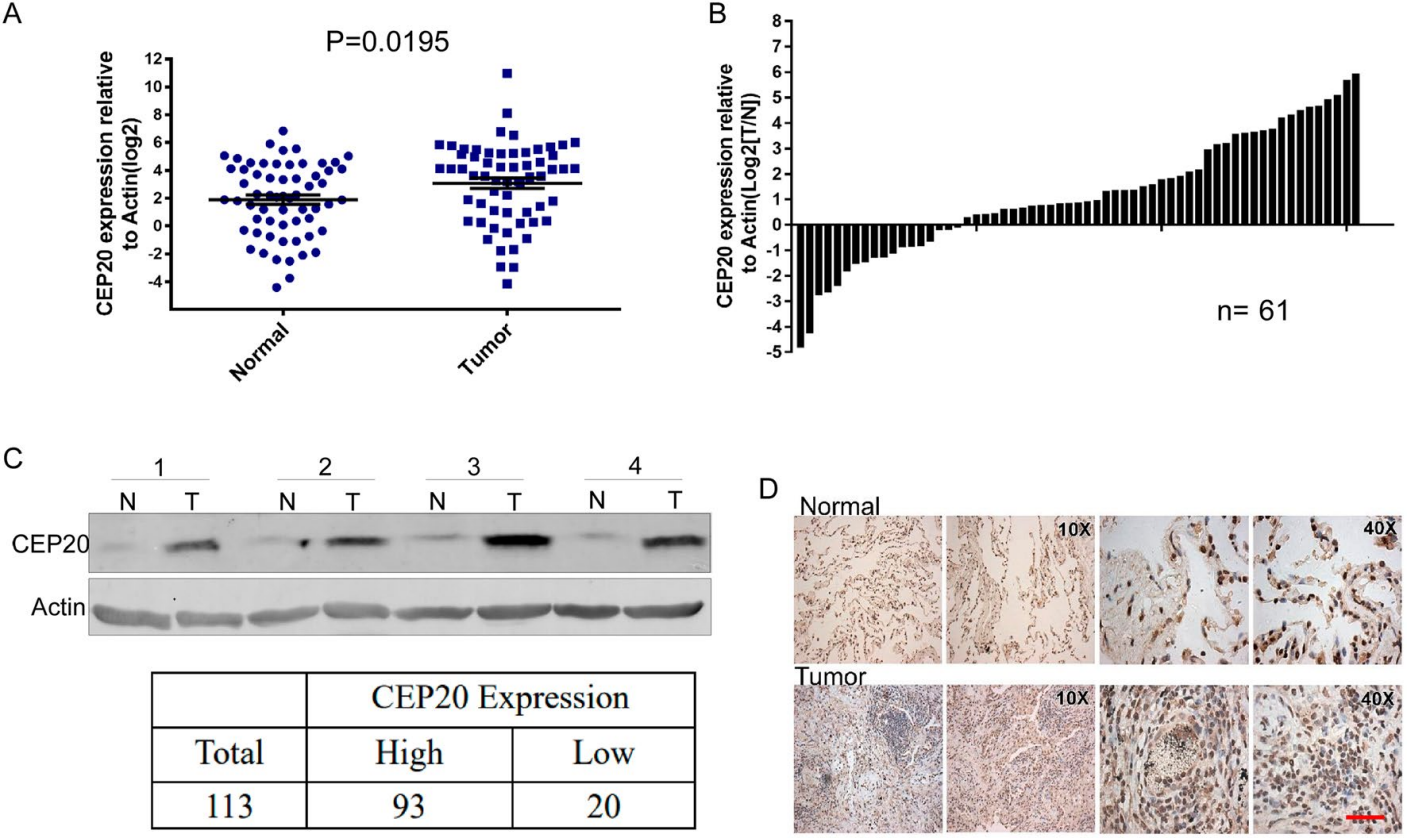
CEP20 is highly expressed in human non-small cell lung cancer tissues. (**A** and **B**) The mRNA expression of CEP20 in non-small cell lung cancer tissues compared to the matched adjacent normal tissues. Actin was used as the internal reference. n = 61, P = 0.0195. (**C**) The protein expression of CEP20 by western blotting in non-small cell lung cancer tissues compared to the matched adjacent normal tissues. Actin was used as the internal reference. Uncropped immunoblots were shown in Supplementary Fig. S8A. (**D**) Representative images of CEP20 staining in non-small cell lung cancer tissues and matched adjacent normal tissues evaluated by an immunohistochemical analysis. Scale bars: 100 μm.

To examine the expression level of CEP20 in NSCLCs, we collected tumor tissues and paired para-carcinoma tissues of patients with NSCLC from the Zhejiang Cancer Hospital. First, we analyzed the mRNA levels of CEP20 in 61 pairs of tumor and para-carcinoma tissues and found a significant upregulation in the tumor samples compared to the para-carcinoma controls, consistent with the results of gene microarray and RNA-seq analyses based on NSCLC samples in the GEO and TCGA databases (Figs. 1A, B and S1A–C). Therefore, we expanded the sampling and extracted the proteins from the pairs of tissues described above to further validate the CEP20 protein level, which may play a critical role in various biological processes. Western blot analysis of 113 pairs of tissues revealed a significantly higher level of CEP20 protein in 93 tumor tissues compared to their matched para-carcinoma tissues (Fig. 1C). Additionally, Immunohistochemistry (IHC) analysis confirmed the overexpression of CEP20 protein in NSCLC tissues (Fig. 1D). Collectively, these results indicated that CEP20 might be a potential key regulator associated with NSCLC tumorigenesis and progression.

Next, we classified the samples into two groups based on the expression level of the CEP20 protein. Statistical analysis of patients’ clinical data demonstrated a significant correlation between CEP20 protein level and tumor invasion, lymphatic metastasis, and distant metastasis (Table 1). These findings further suggest that high expression of CEP20 may be associated with metastasis of lung cancer.

**Table 1:** Clinical characteristics of lung cancer patients according to CEP20 expression.

Characteristics	Total	CEP20 expression		
		High	Low	*P* value
Classification				0.27
Adenocarcinoma	41	31	10	
Squamous	38	33	5	
Gender				0.4276
Male	59	49	10	
Female	20	15	5	
Age (years)				0.3709
< 65	45	38	7	
≥ 65	34	26	8	
Tumor size				**0.0326**
T1	11	11	0	
T2	31	21	10	
T3–4	37	32	5	
Lymph nodes				**0.0462**
N0	24	17	7	
N1	44	36	8	
N2	10	9	1	
N3	2	2	0	
Distant metastasis				**0.0122**
M0	59	44	15	
M1	20	20	0	

Significant values are in [bold].

### Downregulation of endogenous CEP20 inhibits NSCLC cell proliferation

**Figure 2:**
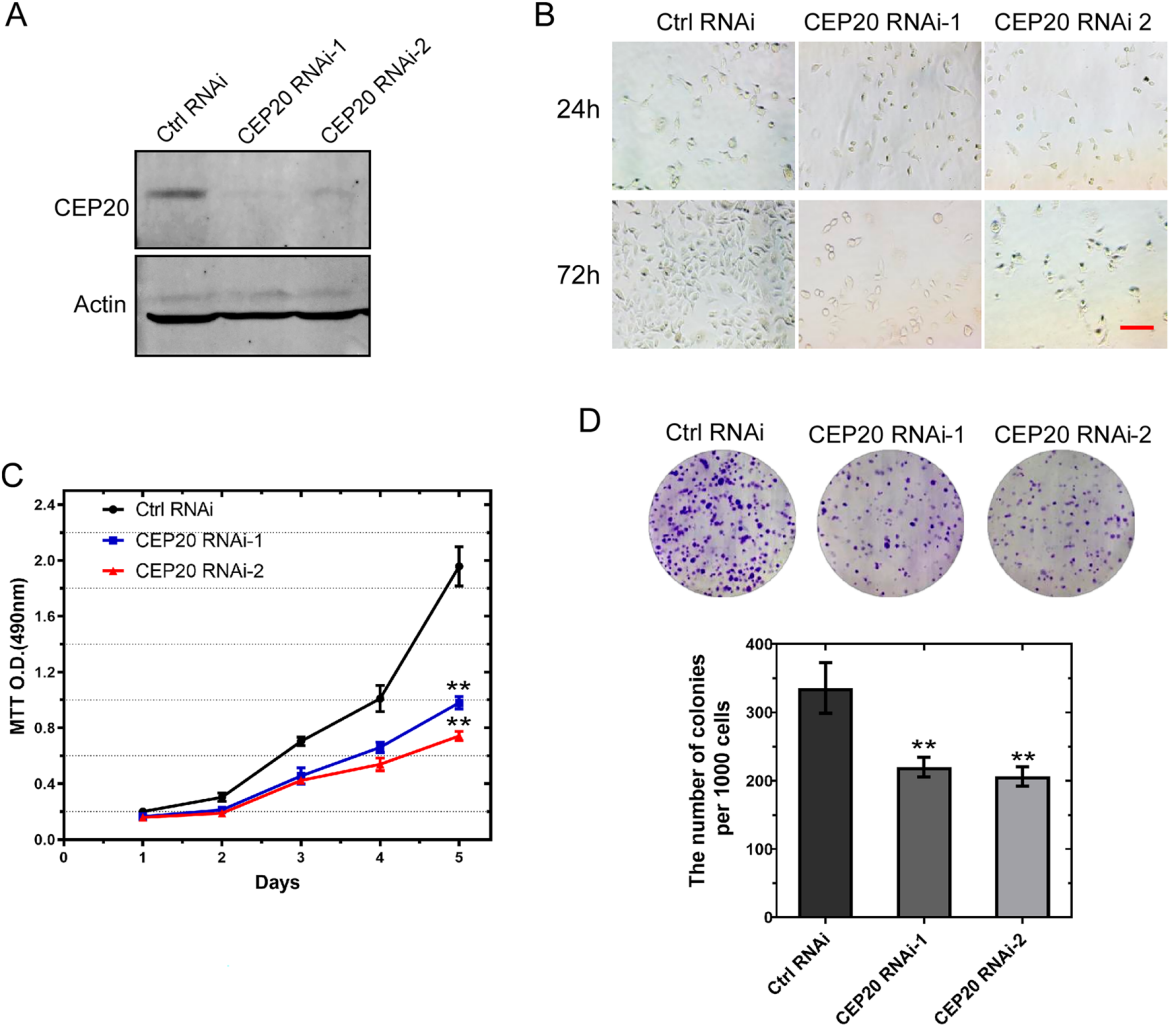
Downregulation of endogenous CEP20 inhibits NSCLC cell proliferation in A549. (**A**) The protein expression of CEP20 by western blotting in A549 cell. Actin was used as internal reference. Uncropped immunoblots were shown in Supplementary Fig. S8B. (**B**) The cell growth was depicted in A549 cell transfected with ctrl RNAi, CEP20 RNAi-1 or CEP20 RNAi-2, respectively. Scale bars: 100 μm. (**C**, **D**) The MTT and colony formation assays were performed in A549 cell transfected with specific ctrl RNAi, CEP20 RNAi-1 or CEP20 RNAi-2, respectively. Bars indicate the mean ± s.d of three independent replicates. *** *p* < 0.001, student’s *t*-test.

Given the previous findings that *CEP20* was overexpressed in NSCLC tissues, and higher level of CEP20 protein was associated with the tumor invasion and metastasis, we further aimed to explore whether CEP20 protein downregulation could influence the tumor’s malignant behaviors, including proliferation, metastasis, and invasion. We utilized RNA interference to downregulate CEP20 in lung adenocarcinoma cell lines A549 and H1299 and lung squamous cell carcinoma cell lines H226 and H520. Western blotting confirmed the efficient knockdown of CEP20 (Figs. 2A, S2A, S4A, and S5A). Our time-course observation of cell growth revealed a significant impairment of the proliferative activity in the CEP20 downregulated group (Fig. 2B). Furthermore, MTT and colony formation assays showed that the downregulation of endogenous CEP20 significantly inhibited A549 and H1299 cell proliferation compared with the control group (Figs. 2C, D, S2B, and C). The same results were also showed in lung squamous cell carcinoma cell lines H226 and H520 (Fig. S4B, C, S5B and C). These findings indicate that CEP20 is critical for NSCLC cell proliferation.

### Downregulation of endogenous CEP20 inhibits NSCLC cell migration and invasion

**Figure 3:**
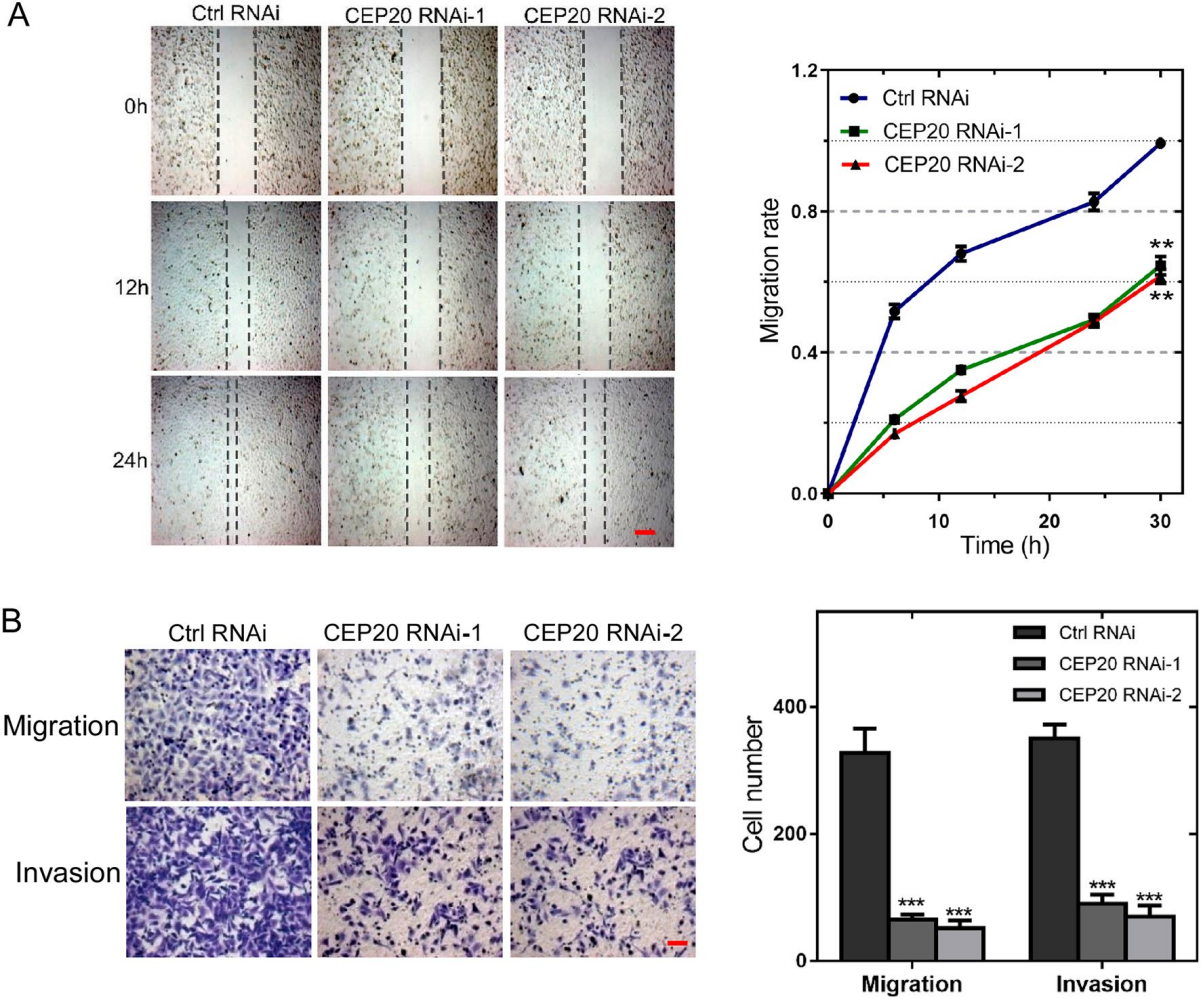
Downregulation of endogenous CEP20 inhibits NSCLC cell migration and invasion. (**A**) The wound-healing assays showed the migration of the ctrl RNAi or specific CEP20 RNAi-1 or CEP20 RNAi-2. The dashed lines indicate the wound edges. Scale bar, 100 μm. The distance of the wound was measured by ImageJ software, and the data are presented as the mean ± standard deviation. (**B**) Transwell assays showed the migration of the ctrl RNAi or specific CEP20 RNAi-1 or CEP20 RNAi-2 and dyed with crystal violet staining. Scale bar, 50 μm. The quantitative data of the randomly selected fields are expressed as the mean ± standard deviation. ** *p* < 0.01, *** *p* < 0.001, student’s* t*-test.

Next, we validated the NSCLC cell motility upon altering CEP20 levels. Wound healing assay revealed a significant reduction in the cell migration capability in CEP20 knockdown A549, H1299, H226 and H520 cells (Figs. 3A, S3A, S4D and S5D). Additionally, we performed a Transwell cell migration assay and Invasion experiment to further identify the role of CEP20 in NSCLC cell motility. Transwell cell migration assay indicated a significantly decrease in cell migration upon CEP20 knockdown compared to the control group (Figs. 3B and S3B). Meanwhile, the invasion experiment revealed a significant reduction in invasion activity in CEP20 knockdown cells compared with the control group (Figs. 3B, S3B, S4E, F, S5E and F). These results suggest that CEP20 plays crucial roles in NSCLC cell motility, including migration and invasion.

### Exogenous CEP20 could rescue the impaired NSCLC cell proliferation and mobility induced by knockdown of CEP20

**Figure 4:**
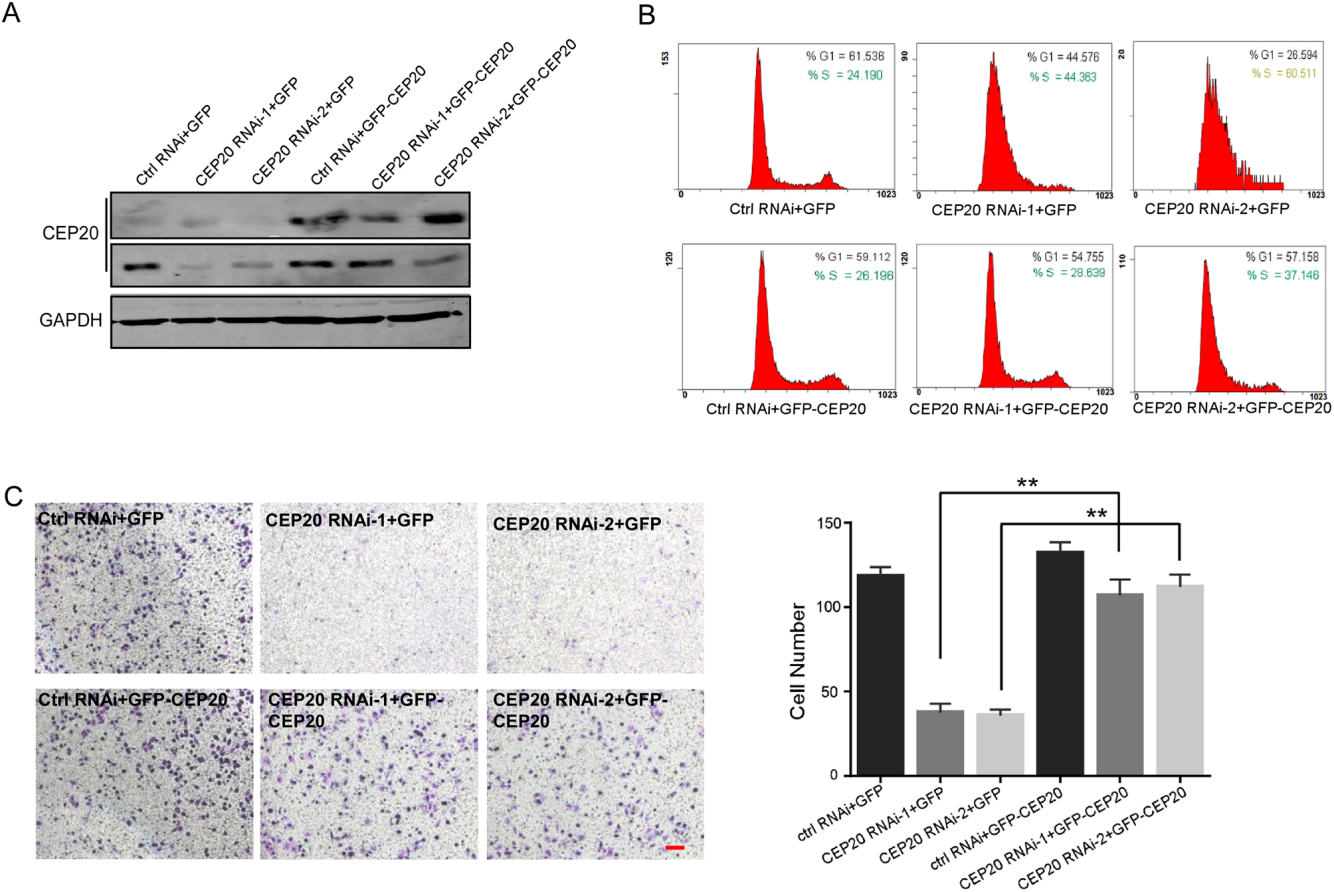
Depletion of CEP20 inhibits cell proliferation and cell migration. A549 cells were transfected with the indicated RNA interference and plasmids and then subjected to the following assays. (**A**) Western blotting revealed the expression level of endogenous CEP20 and ectopic GFP-CEP20 in A549 cells. GAPDH was used as an internal control. Uncropped immunoblots were shown in Supplementary Fig. S8C. (**B**) Flow cytometric analysis showed the cell proliferation transfected with the indicated treatments. (**C** and **D**) Transwell analysis exhibited the migration of the indicated cells. Quantitative data of randomly selected fields (n > 3) were shown. Bar, 50 μm. **P < 0.01, one-way ANOVA.

To further investigate the role of CEP20 in NSCLC cell proliferation and mobility, we performed a rescue experiment by overexpressing CEP20 using pEGFP-CEP20 expressing vector in A549 cells. We first downregulated the CEP20 mRNA level using CEP20 RNAi-1 and RNAi-2, then rescue experiments were performed using the ectogenic pEGFP-CEP20, quantified by western blotting analysis (Fig. 4A). Flow cytometry analysis demonstrated that the endogenous CEP20 knockdown cells were arrested in the S stage of the cell cycle, while overexpression of pEGFP-CEP20 rescued the cell cycle distribution to a relatively normal pattern (Fig. 4B). Accordingly, the Transwell experiment also confirmed that the rescued cell showed enhanced motility compared to cells treated with only CEP20 RNAi, similar to the motility of the untreated control group (Fig. 4C). These findings confirmed that the downregulation of endogenous CEP20 protein affects NSCLC cell proliferation and mobility.

### CEP20 promotes malignant behavior of NSCLC cells by depolymerizing microtubules

**Figure 5:**
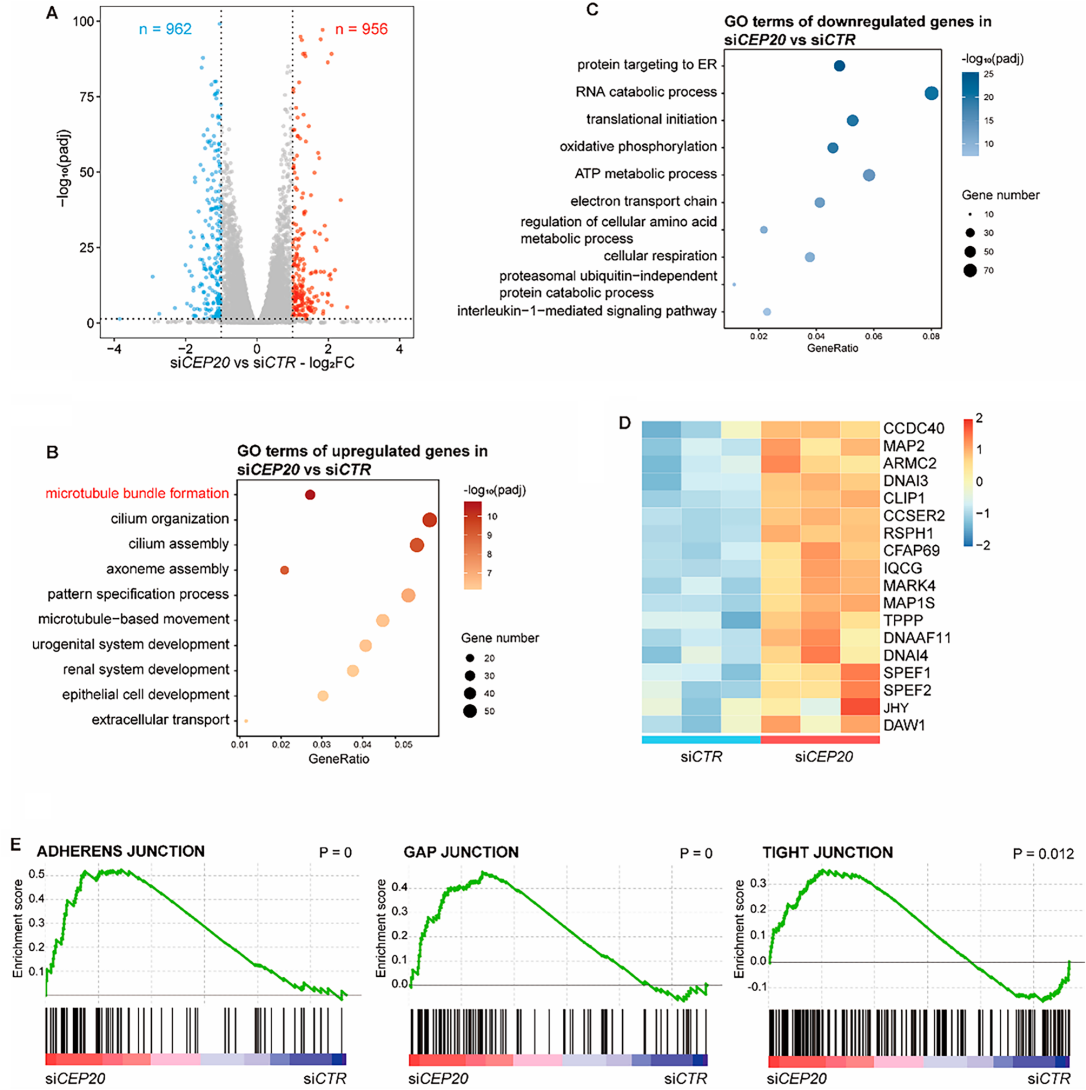
CEP20 knockdown promotes high expression of microtubule bundle formation and cell adhesion related genes. (**A**) Volcano plot showing the differentially expressed genes in si*CEP20* vs si*CTR* samples. Red dots: upregulated genes in si*CEP20* samples, blue dots: downregulated genes in si*CEP20* samples. (**B**, **C**) Bubble plots of GO terms enriched in up- and downregulated genes in in si*CEP20* samples. Point sizes represent the gene numbers in each term, orange and blue gradients represent the − log_10_ (adjusted *P* values). (**D**) Expression heatmap of microtubule bundle formation related genes in si*CTR* and si*CEP20* samples. (**E**) GSEA of adherens junction, gap junction and tight junction pathways in si*CEP20* versus the si*CTR* samples.

To further investigate the mechanism by which CEP20 promotes the malignant behavior of NSCLC cells, we performed RNA sequencing (RNA-seq) of A549 CEP20-knockdown and control cells (Fig. S6A). Differential expression analysis identified 956 upregulated and 962 downregulated genes in response to CEP20 knockdown (Fig. 5A). Gene Ontology (GO) analysis determined that upregulated genes were enriched in processes related to microtubule bundle formation, cilium organization, and organ development, consistent with our previous findings that CEP20 is a crucial regulator in ciliogenesis (Fig. 5B). On the other hand, downregulated genes were enriched in processes related to protein targeting to ER, translational initiation, and oxidative phosphorylation. All these biological processes are closely related to the development of malignant tumors, indicating that the high expression of CEP20 promotes protein synthesis and energy metabolism in NSCLC cells, leading to further aggravation of malignant tumor behaviors (Fig. 5C).

To further identify the specific mechanism of CEP20 in NSCLC cells, we divided NSCLC tumor samples (LUAD and LUSC samples, n = 1014) in the TCGA database into three groups based on CEP20 expression: relatively high (CEP20-high, n = 253); relatively low (CEP20-low, n = 253), and medium (CEP20-median, n = 508). Differential expression analysis of CEP20-high and CEP20-low groups identified 572 significantly upregulated and 1112 significantly downregulated genes (Figure S6B). Moreover, GO analysis demonstrated that downregulated genes were related to cilium movement, extracellular matrix organization, and microtubule bundle formation (Figure S4C). Consistent with our RNA-seq results, we found that ‘microtubule bundle formation’ was in the top GO terms of CEP20 knockdown or CEP20-low groups and that these microtubule bundle formation-related genes were significantly highly expressed (Figs. 5B, D, and S6C–D). Also, the significantly activated Kyoto Encyclopedia of Genes and Genomes (KEGG) pathways included adherens junction, gap junction, and tight junction, suggesting that CEP20 reduces cell adhesion by depolymerizing microtubules in NSCLC cells (Fig. 5E). Taken together, our results demonstrate that CEP20 modulates the expression of many essential genes critical for microtubule bundle formation and is essential for the malignant behavior of NSCLC cells.

**Figure 6:**
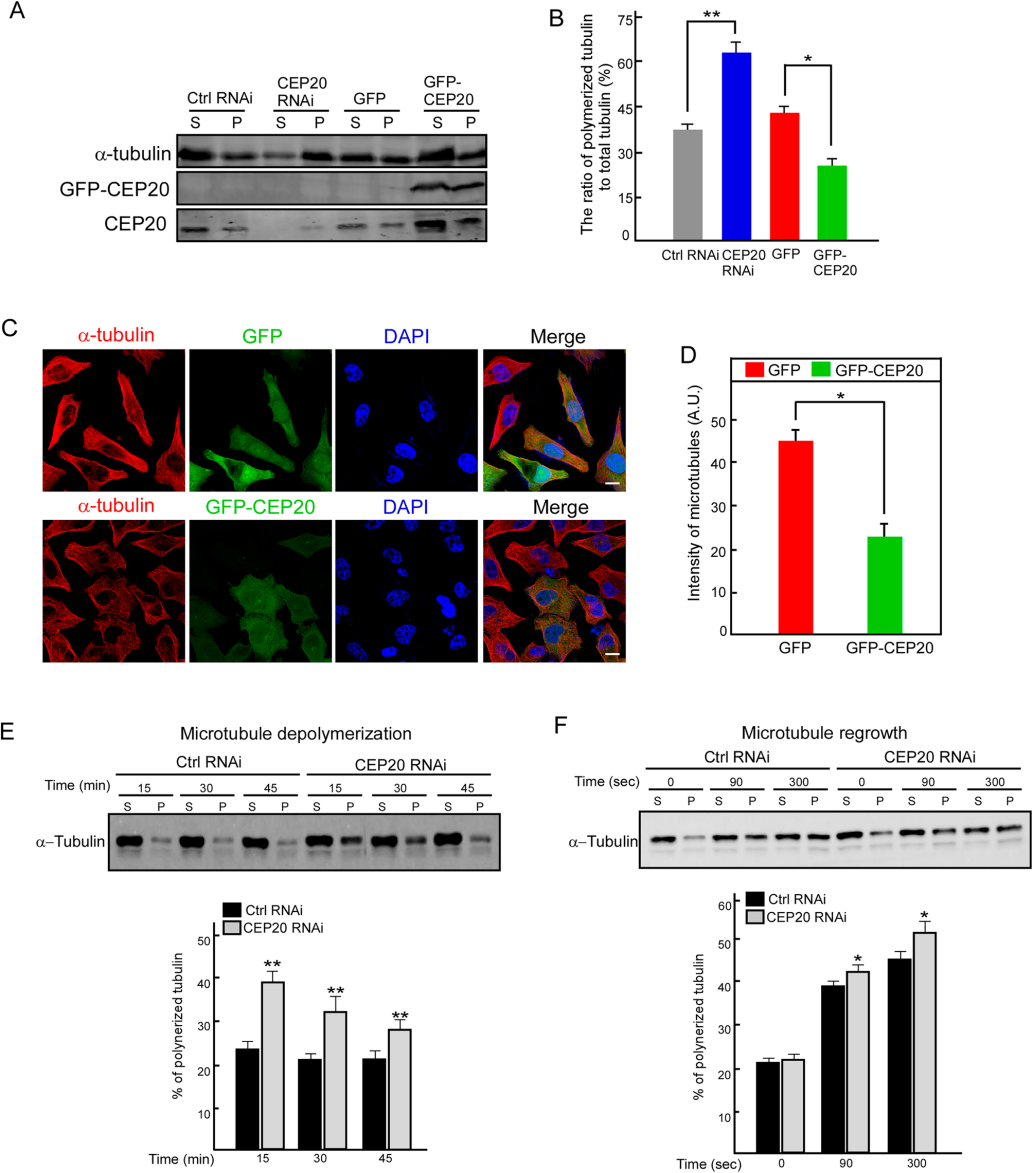
Depletion or overexpression of CEP20 changes the status of microtubule polymerization in A549 cells. (**A** and **B**) Lysates from A549 cells transfected with the indicated plasmids were subjected to ultracentrifugation. The supernatant (S) and pellet (P) fractions were then processed for Western blotting with anti-α-tubulin and CEP20 antibodies. Uncropped immunoblots were shown in Supplementary Fig. S8D. The intensities of the bands were quantified by ImageJ. (**C** and **D**) A549 cells transfected with GFP or GFP-CEP20 were processed for immunoflourescence assay with anti-α-tubulin antibody. DNA was stained with DAPI (blue). Bar, 10 μm. The average intensity of microtubules in cells (n = 40) transfected with GFP or GFP-CEP20 was determined by ImageJ. Quantitative data of microtubule intensities are presented as mean ± SD. (**E** and **F**) Lysates from A549 cells treated with nocodazole and CEP20 depletion were subjected to ultracentrifugation. The supernatant (S) and pellet (P) fractions were then processed for Western blotting with anti-α-tubulin. Uncropped immunoblots were shown in Supplementary Fig. S8E and F. Quantitative data of polymerized tubulin are presented as mean ± SD. *P < 0.05 and **P < 0.01, student’s *t*-test.

Based on our previous results, we first proposed CEP20 as an important activator in NSCLC cell invasion and migration by regulating the dynamic assembly of microtubules. Next, to verify the role of CEP20 in regulating microtubules, we employed co-sedimentation assays to quantify the soluble and polymerized tubulin contents in cells depleted of or overexpressing CEP20. Our results show that the ratio of polymerized tubulin to total tubulin content significantly increased in cells depleted of CEP20 and decreased in cells with CEP20 overexpression compared with the control group (Fig. 6A and B). Furthermore, we expressed GFP-CEP20 in HeLa cells and performed immunofluorescence experiments. We found that the microtubule intensity in cells with ectopic GFP-CEP20 was significantly decreased compared with the GFP control cells (Fig. 6C and D), indicating that CEP20 overexpression may inhibit microtubule formation and destabilize microtubules in mammalian cells. Furthermore, we employed co-sedimentation assays to quantify the soluble and polymerized tubulin contents in cells treated with nocodazole and CEP20 depletion. Our results showed that the ratio of polymerized tubulin to total tubulin content significantly increased in cells depleted of CEP20 (Fig. 6E and F). We also investigated cellular microtubule dynamics in cells treated with nocodazole and CEP20 depletion. During the microtubule regrowth after transient treatment with nocodazole, CEP20 depletion significantly promoted microtubule assembly (Fig. S7A and B).

Taken together, these results further confirm our hypothesis that CEP20 may play inhibitory roles in microtubule dynamics in mammalian cells, thereby regulating NSCLC cell invasion and migration. Therefore, CEP20 is a promising potential therapeutic target for NSCLC, and our results provide new strategies and directions for the development of clinical therapy.

## Discussion

Lung cancers are a highly prevalent malignancy with high morbidity and mortality globally^1^. Unfortunately, many patients are diagnosed at an advanced stage, resulting in poor outcomes. Conventional chemotherapy has limited efficacy in improving the prognosis of late-stage patients, and resistance can develop with EGFR-TKIs^24^. Consequently, novel mechanisms of targeted treatment are urgently needed. As microtubule dynamics are crucial for tumor cell mitosis and proliferation^9,10^, recent studies have promoted anti-microtubules drugs as one of the most effective malignancy treatments^14,15^.

In this study, we systematically illustrated the regulatory role of CEP20 in tumorigenesis and the progression of NSCLC cells. Our findings suggest that CEP20 is overexpressed in human NSCLC cells and is involved in the regulation of NSCLC cell proliferation and motility by regulating microtubule dynamics. Therefore, this novel negative regulator of microtubule assembly might provide potential direction for NSCLC treatment.

Our analysis of public databases and patient samples revealed that the mRNA and protein expression of CEP20 in the tumor samples is higher than in the adjacent normal samples. Moreover, higher CEP20 protein levels were associated with increased lymph nodes and distant metastasis, indicating that CEP20 upregulation is associated with a worse prognosis for patients with NSCLC. The cellular level analysis demonstrated that downregulation of intrinsic CEP20 inhibited cell proliferation, migration, and invasion in NSCLC cell lines A549 and H1299, suggesting a potential role of CEP20 in NSCLC progression and metastasis. Our future experiments will aim to better understand the influence of CEP20 in NSCLC tumorigenesis and metastasis using tumor xenograft models.

Our sequencing data of CEP20-knockdown A549 cells revealed that CEP20 might be involved in NSCLC tumorigenesis and progression by regulating microtubule dynamics. Our previous research has shown that CEP20 is a centrosomal protein and a newly identified microtubule-associated protein involved in ciliogenesis, indicating its potential as a new target for lung cancer treatment. Our future experiments will also examine the protein structure of CEP20 and its microtubule-binding domains using protein crystallization and cryo-electron microscopy (cryo-EM). This will enable us to identify and select small molecular inhibitors that target CEP20 and further investigate the molecular mechanisms underlying the regulatory role of CEP20 on NSCLC cell proliferation, migration, tumorigenesis, and invasion, as well as its effect on microtubules.

## Conclusion

Overall, our findings provide a new target for targeted therapy of non-small cell lung cancer, and the potential utility of CEP20 in lung cancer diagnosis and treatment deserves further exploration at the molecular, cellular, animal model, and clinical pathology dimensions.

## Materials and methods

### Ethics approval statement

All the human non-small cell lung cancer samples were obtained from Zhejiang Cancer Hospital with informed consent. This study was conducted in accordance with the Declaration of Helsinki (as revised in 2013). The Ethics Committee in Clinical Research (ECCR) of The Cancer Hospital of the University of Chinese Academy of Sciences (Zhejiang Cancer Hospital) approved this retrospective study (IRB-2022-268). The authors are accountable for all aspects of the work in ensuring that questions related to the accuracy or integrity of any part of the work are appropriately investigated and resolved.

### Patient consent statement

All patients signed an informed consent approved by the institutional Review Board.

### Quantitative RT–PCR assay

Quantitative real-time–polymerase chain reaction (qRT-PCR) was performed using 2 × Synergy Brands Inc (SYBR) Green (Servicebio) on the Roche LightCycler480 II RT-PCR Detection System. The relative CEP20 expression was quantified by RT-PCR, and Actin was used as an internal reference. All the reactions were triplicated and were calculated using the comparative threshold method (${2}^{{ - \Delta \Delta {\text{C}}_{{\text{t}}} }}$).

### Western blotting

Cell lysates or microtubule pellets were subjected to western blotting analysis with anti-CEP20, Actin, or GAPDH antibodies (Sigma, St Louis, MO, USA). The blots were probed with either Alexa Fluor 680 or IRDye 800-conjugated secondary antibodies and detected using the Odyssey system (LI-COR Biosciences, Lincoln, NE, USA). The uncropped immunoblot images are presented in Fig. S8.

### Cell culture

A549 and H1299 cells were cultured in a complete DMEM (10% FBS included) medium with 5% CO_2_ at 37 °C. H226 and H520 were cultured in a complete RPMI 1640 (10% FBS included) medium with 5% CO_2_ at 37 °C. Cells were split at approximately 80% confluence by first aspiring the medium, followed by washing with preheated sterile 1 × PBS buffer thrice. Trypsin was given for 1 min to induce cell detachment at 37 °C, then terminated by adding an appropriate volume of the medium. The cell mixture was transferred into a 15 mL Falcon tube and dissociated to form a single-cell suspension by pipetting up and down. An appropriate volume of suspension was added to a new plate for continuous culture.

### RNA interference and cell transfection

The cells were cultivated to the logarithmic growth phase and passaged the day before transfection. When cells reached 20–30% confluence, Lipofectamine RNAiMAX and the siRNAs (CEP20 RNAi-1 5′-ACCACTAATGTTTGTAGAATT-3′ CEP20 RNAi-2 5′-ATGGATGACCACCTAAGAATT-3′) were diluted with DMEM (FBS free) according to the corresponding transfection system. Each dilution was incubated for 5 min, mixed well, and incubated for another 20 min at room temperature. After adding the mixture into corresponding groups, cells were cultured for 6 h in a 5% CO_2_ incubator at 37 °C. Subsequently, the medium was replaced with complete DMEM containing 10% FBS. After 48–72 h of transfection, the cells were observed under a fluorescent microscope to evaluate their condition and transfection efficiency for further analysis.

### MTT (3-(4,5-dimenthylthiazol-2-yl)-2,5-diphenylterazolium bromide) cell viability assay

A549 and H1299 cells were transfected and subsequently cultured for 48 h. Then cells were evenly passage to 96-well plates with 2 × 10^3^ cells per well. Cultured for 24, 48, 72 and 96 h, cells were added with 20 μL/well MTT solution (5 mg/ml, Sigma, St. Louis, MO, USA) and incubated for 4 h at 37 °C. Then the medium was discarded and added 150 μL of dimethyl sulphoxide (DMSO) (Sigma, St. Louis, MO, USA), the cell proliferation was analyzed by measuring the absorption at 490 nm. Cell growth curves were depicted by Graphpad Prism 9 software.

### Colony formation assay

Cells were plated on 12-well plates (200 cells per well). The cell culture medium was changed every 2 days. After 2 weeks, the cells were fixed with 4% paraformaldehyde for 15 min, then washed 3 times by phosphate buffered solution (PBS), and dyed with crystal violet staining solution for 30 min.

### Wound healing assay

Cells were transfected and subsequently cultured until they reached 100% confluence. The cells were then starved overnight using a bare medium (DMEM or RPMI 1640 with no glucose or FBS). Mechanical scratching (wound) was performed manually with a pipette tip (10 μl), and the medium was replaced with DMEM or RPMI 1640 containing 1% FBS. Cells were imaged every 12 h. It is important to note that the same area of the wound was imaged consistently across time points.

### Transwell cell migration assay

Cells were transfected following the protocol described above. After 48 h of transfection, cells were starved overnight using a bare medium (DMEM or RPMI 1640 with no glucose or FBS). Subsequently, cells were trypsinized and counted, and 80,000 starved cells were resuspended in DMEM or RPMI 1640 containing 1% FBS and added to the upper chamber of Transwell inserts. The lower chamber was filled with 600 μl of complete DMEM or RPMI 1640 containing 10% FBS. The cells were then cultured for 4 h at 37 °C with 5% CO2. After incubation, the transwell chambers were taken out and fixed with 4% PFA for 20 min at room temperature. The inserts were stained with crystal violet for 20 min, then washed for 5 min each thrice. Residual non-migratory cells from the upper chamber were wiped off with a swab, while the migratory cells were counted and imaged under a microscope.

### Immunofluorescence

A549 cells grown on coverslips were fixed with cold methanol (− 20 °C), stained with anti-CEP20, α-tubulin antibodies (Sigma, St Louis, MO, USA) for 2 h at room temperature, and incubated with either Cy3-conjugated anti-mouse IgG or FITC-conjugated anti-rabbit IgG secondary antibody (Jackson ImmunoResearch) for 40 min. DNA was stained with DAPI (Sigma). Finally, the mounted coverslips were analyzed by confocal fluorescence microscopy (LSM510, Zeiss).

### Cellular microtubule depolymerization and regrowth

For the cellular microtubule depolymerization assay^21^, A549 cells were treated with 5 μM nocodazole for the indicated times, and then centrifuged at 100 000 g for 20 min at 25 °C. For the cellular microtubule regrowth assay, A549 cells grown on coverslips were incubated with 5 μM nocodazole for 3 h to depolymerize microtubules, and then carefully washed out to remove nocodazole followed by fixation at the indicated times. All cells were stained with mouse anti-α-tubulin primary antibody and Cy3-conjugated anti-mouse IgG secondary antibody. The coverslips were then mounted and imaged by confocal microscopy (NIKON, Tokyo, Japan). The astral length of microtubules within the region of interest were quantified using ImageJ software (Fiji, NIH). Data are expressed as mean ± s.d. and analyzed by student’s t-test. The supernatant and pellet fractions were collected separately and analyzed by western blotting with anti α-tubulin.

### RNA extraction and library construction

Total RNA was isolated and purified using TRIzol reagent (Invitrogen, Carlsbad, CA, USA) following the manufacturer’s instructions. The RNA amount and purity of each sample were quantified using NanoDrop ND-1000 (NanoDrop, Wilmington, DE, USA). The RNA integrity was assessed using Bioanalyzer 2100 (Agilent, CA, USA) with a RIN above 7.0 and confirmed by denaturing agarose gel electrophoresis. Poly (A) RNA was purified from 1 μg total RNA using Dynabeads Oligo (dT) 25-61005 (Thermo Fisher, CA, USA) with two rounds of purification. The purified poly(A) RNA was fragmented into small pieces using the Magnesium RNA Fragmentation Module (NEB, e6150, USA) at 94 °C for 5–7 min. The cleaved RNA fragments were then reverse-transcribed using SuperScript™ II Reverse Transcriptase (Invitrogen, cat. 1896649, USA). The resultant cDNA was used to synthesize U-labeled second-stranded DNAs with E. coli DNA polymerase I (NEB, m0209, USA), RNase H (NEB, m0297, USA), and dUTP solution (Thermo Fisher, R0133, USA). An A-base is then added to the blunt ends of each strand, preparing them for ligation to the indexed adapters. Each adapter contained a T-base overhang for ligating the adapter to the A-tailed fragmented DNA. Single- or dual-index adapters are ligated to the fragments, and size selection was performed with AMPureXP beads. After treatment with the heat-labile UDG enzyme (NEB, m0280, USA) to remove the U-labeled second-stranded DNAs, the ligated products are amplified using PCR. The PCR conditions were as follows: initial denaturation at 95 °C for 3 min; 8 cycles of denaturation at 98 °C for 15 s, annealing at 60 °C for 15 s, and extension at 72 °C for 30 s; and final extension at 72 °C for 5 min. The average insert size for the final cDNA library was 300 ± 50 bp. Finally, the 2 × 150 bp paired-end sequencing (PE150) was performed on an Illumina Novaseq™ 6000 according to the manufacturer’s protocol.

### RNA-seq analysis

Raw RNA-seq data were processed using fastp (v0.20.1)^25^ to remove adapter sequences and reads with low sequencing quality. The remaining clean reads were aligned to the human genome (hg38) using HISAT2 software (v2.1.0)^26^ with default parameter settings. Transcript assembly was performed using StringTie software (v2.0)^27^, and expression of transcripts sharing each gene_id was quantified as Transcripts Per Million (TPM). Differential expression analysis was performed using the R package DESeq2^28^ with a threshold of significantly differentially expressed genes set as fold change (FC) > 1.5 or < 0.67 and adjusted *P* value < 0.05. Heatmaps were generated using the R package pheatmap. The Gene Ontology (GO) term and Kyoto Encyclopedia of Genes and Genomes (KEGG) pathway enrichment analyses in current study were done by R package clusterProfiler^29^. Adjusted *p* value < 0.05 was considered as statistically significant. The gene set enrichment analysis (GSEA) was performed by R package enrichplot.

### NSCLC gene-microarray and RNA-seq sample collection

The dataset GSE19804^30^ based on the platform of GPL570 (Affymetrix Human Genome U133 Plus 2.0 Array) containing 30 paired gene-microarray samples of human NSCLC tumor and normal tissues were downloaded from the Gene Expression Omnibus (GEO) database (https://www.ncbi.nlm.nih.gov/geo). The RNA-seq data of NSCLC samples were retrieved from the Cancer Genome Atlas (TCGA) database (https://portal.gdc.cancer.gov/), including 513 lung adenocarcinoma (LUAD) tumor samples, 57 LUAD adjacent normal samples and 501 lung squamous cell carcinoma (LUSC) tumor samples, and 49 LUSC adjacent normal samples. The expression levels of CEP20 were extracted from these datasets, and the NSCLC samples from the TCGA database were classified into three groups based on their CEP20 expression levels: relatively high (CEP20-high, n = 253), relatively low (CEP20-low, n = 253), and medium (CEP20-median, n = 508).

### Statistical analysis

All experiment results are presented as mean ± standard deviation (SD) from 3 independent experiments and showed successful reproducibility. All graphs were generated using GraphPad Prism 9 (64-bit, La Jolla, CA, USA). Two-tailed unpaired t-tests (Student’s t-test) were used to obtain the *p* values. The data are presented as the mean ± standard deviation. *P < 0.05, **P < 0.01, ***P < 0.001.

### Supplementary Information

Supplementary Information 1.

Supplementary Information 2.

Supplementary Information 3.

Supplementary Information 4.

Supplementary Information 5.

Supplementary Information 6.

Supplementary Information 7.

Supplementary Information 8.

Supplementary Information 9.

## Data Availability

The raw RNA-seq data generated in this study have been deposited in the GEO database under accession number GSE218949.
