# Supplementary figures and images for: CEP20 promotes invasion and metastasis of non-small cell lung cancer cells by depolymerizing microtubules
Source: Sci Rep. 2023 Oct 14;13:17484. doi: 10.1038/s41598-023-44754-8 (PMC10576744; doi:10.1038/s41598-023-44754-8)

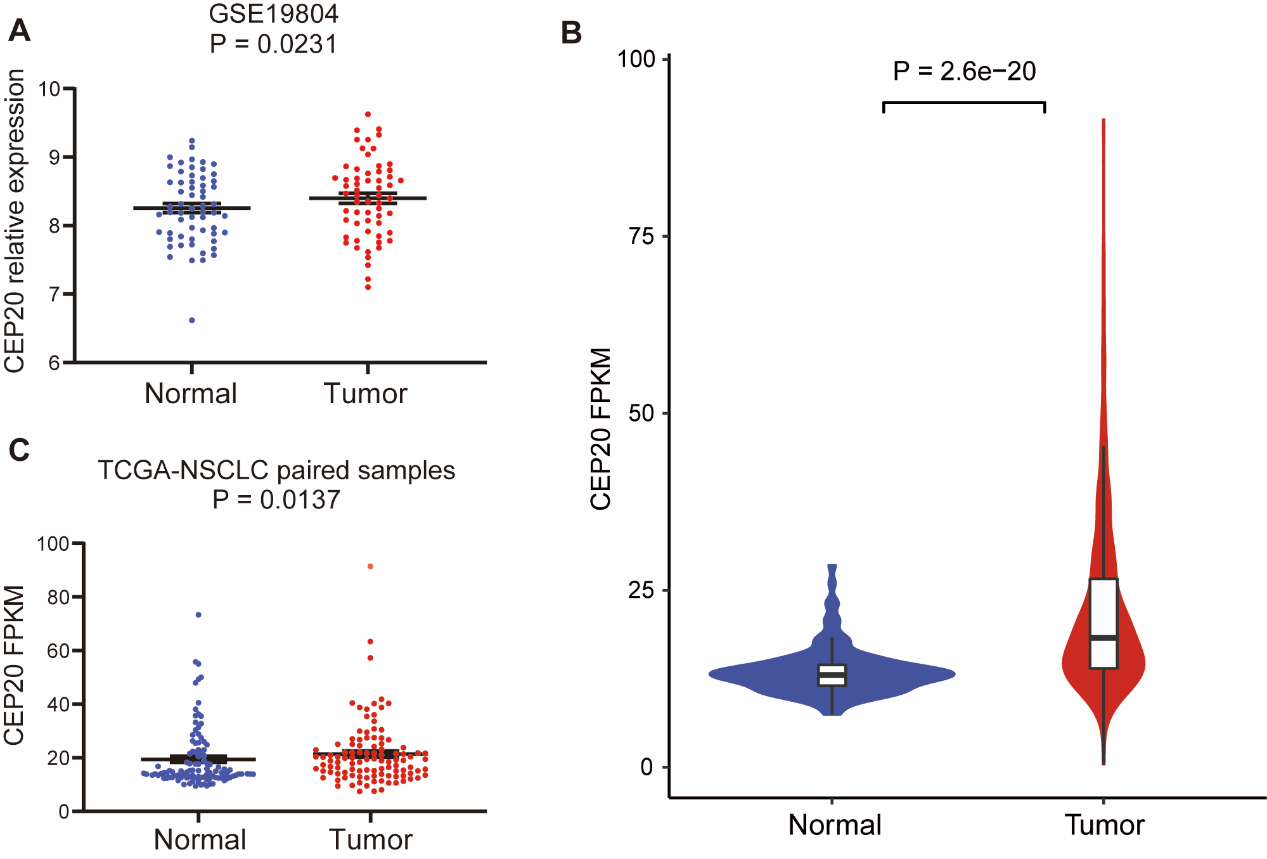

Supplement: Supplementary file 1 — Supplementary Information 1. [file 41598_2023_44754_MOESM1_ESM.tif]

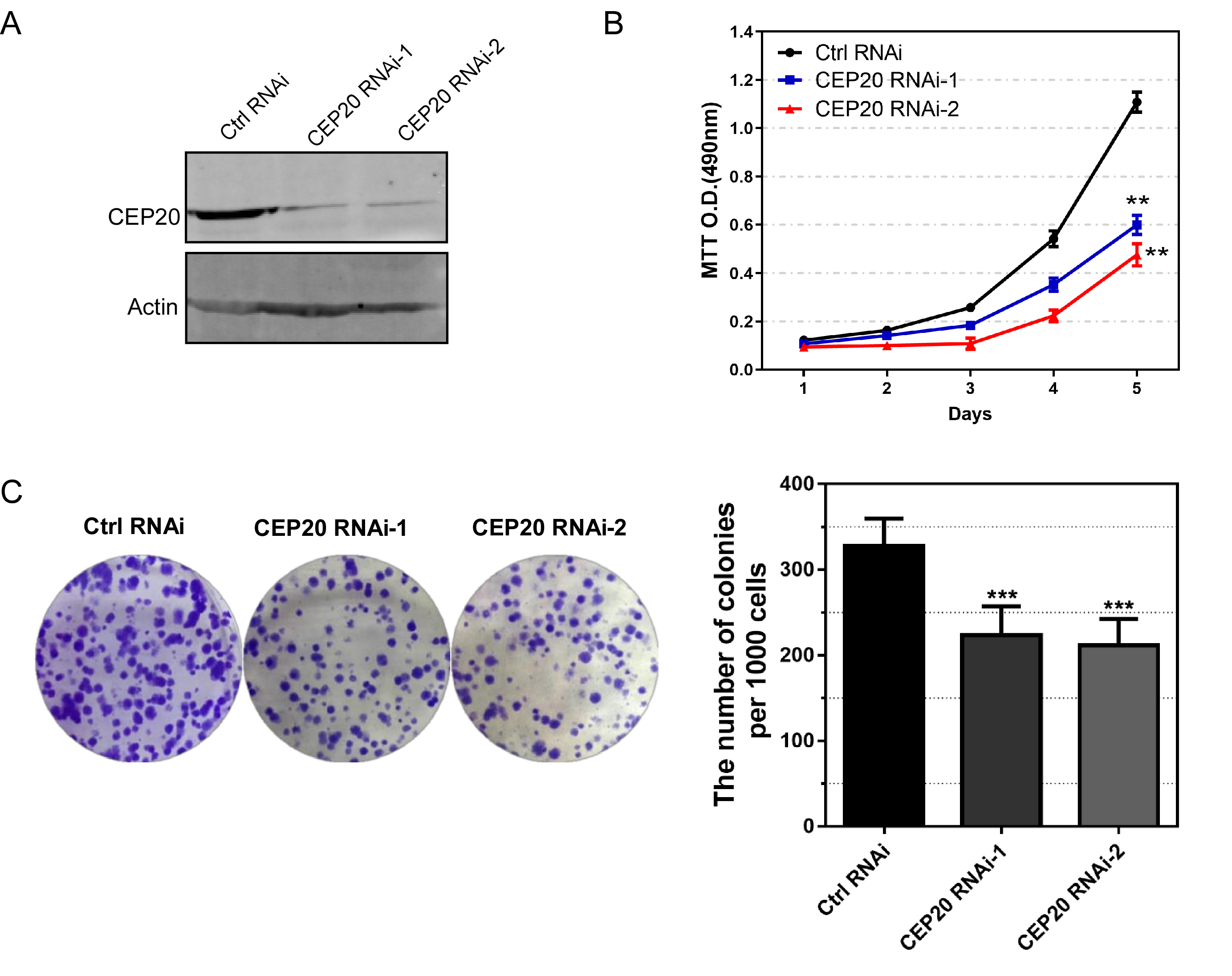

Supplement: Supplementary file 2 — Supplementary Information 2. [file 41598_2023_44754_MOESM2_ESM.tif]

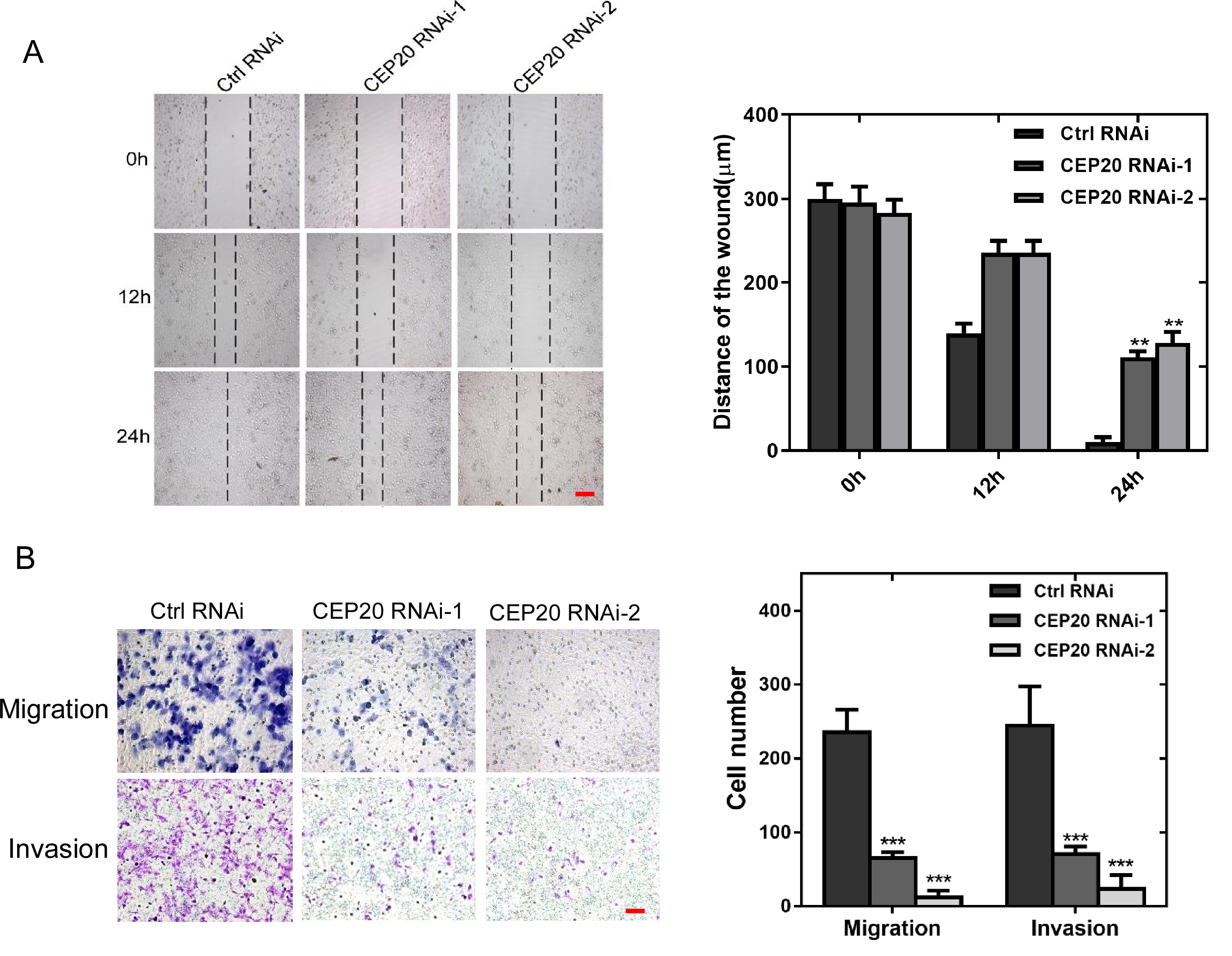

Supplement: Supplementary file 3 — Supplementary Information 3. [file 41598_2023_44754_MOESM3_ESM.tif]

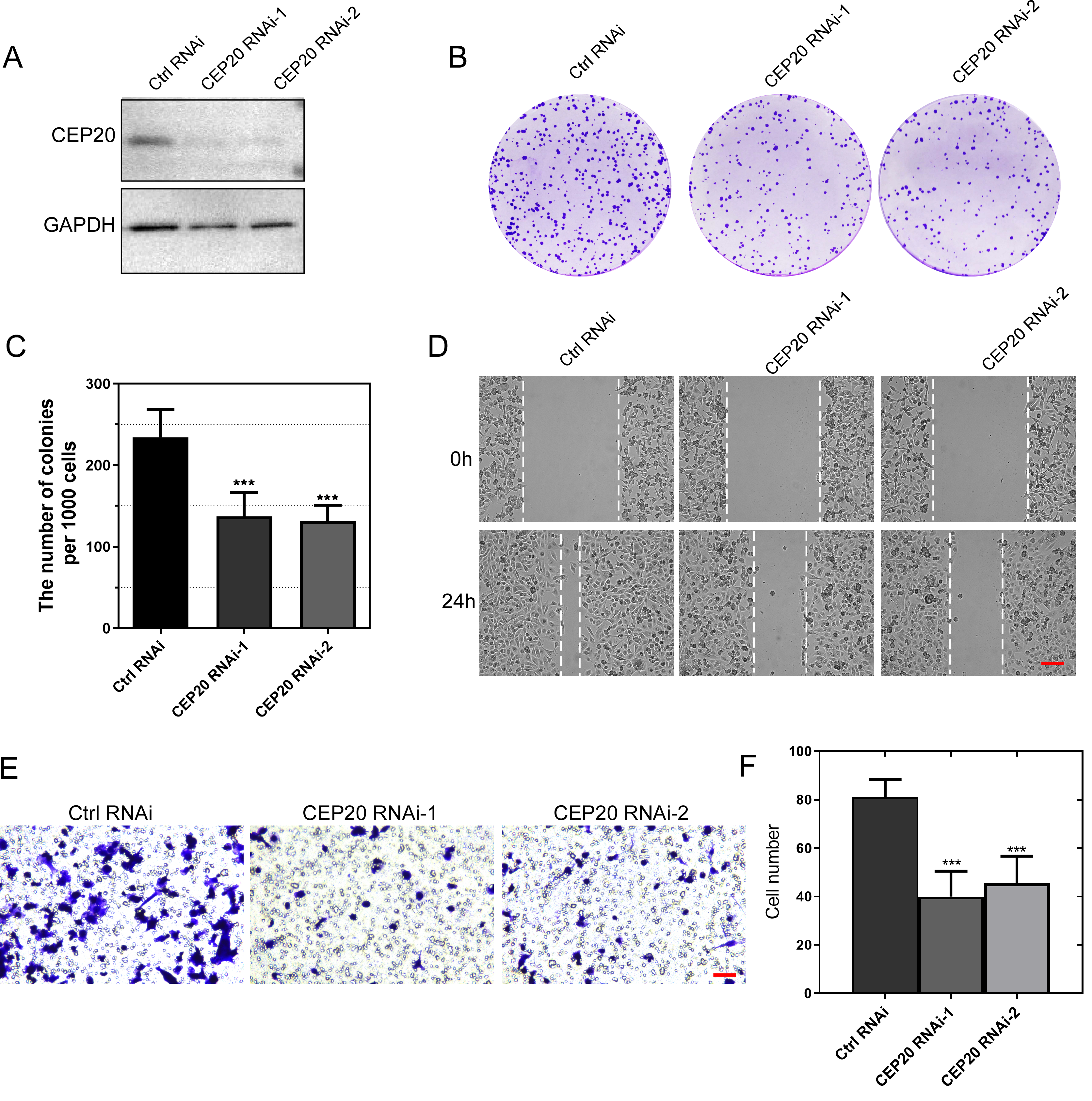

Supplement: Supplementary file 4 — Supplementary Information 4. [file 41598_2023_44754_MOESM4_ESM.tif]

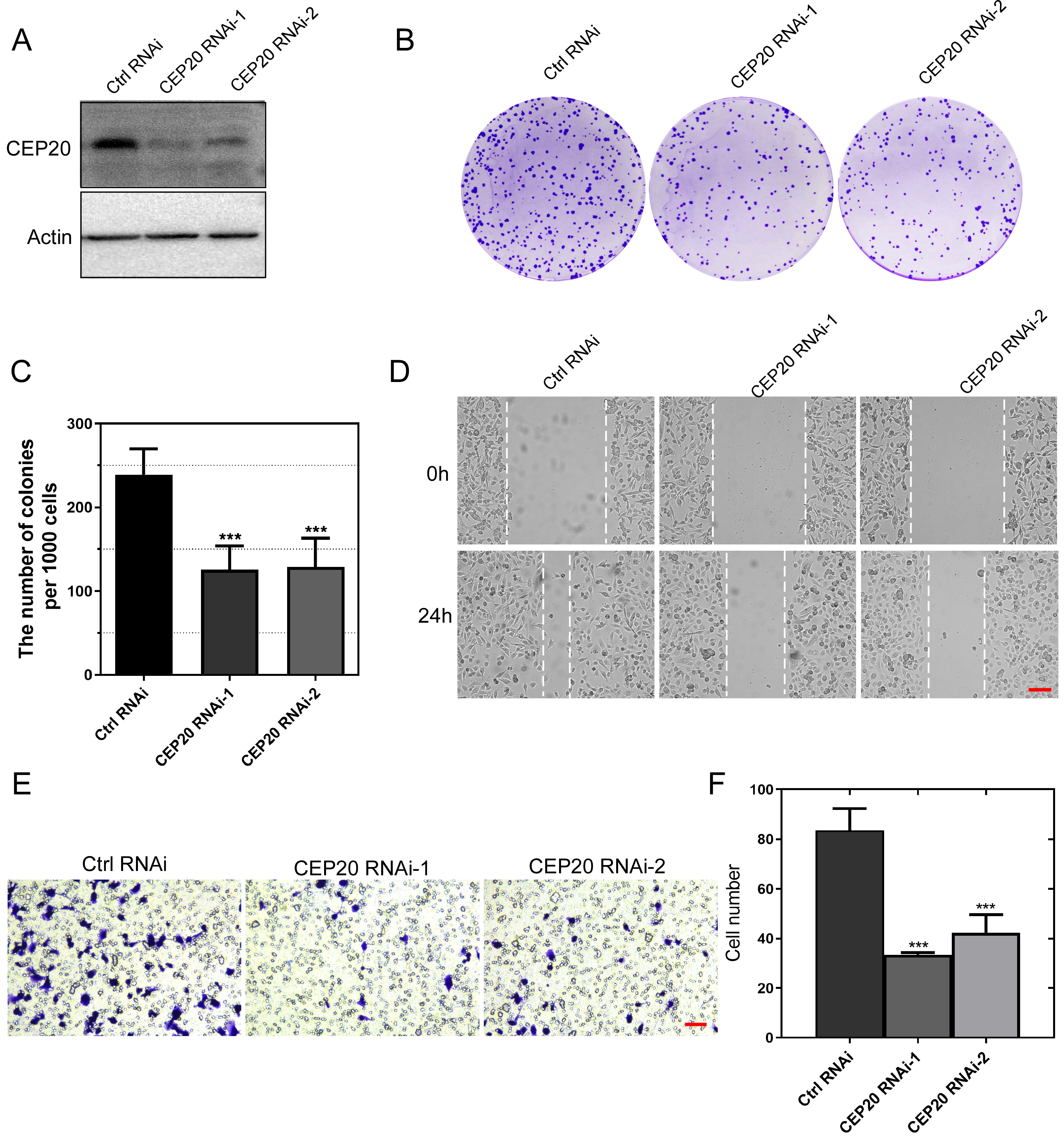

Supplement: Supplementary file 5 — Supplementary Information 5. [file 41598_2023_44754_MOESM5_ESM.tif]

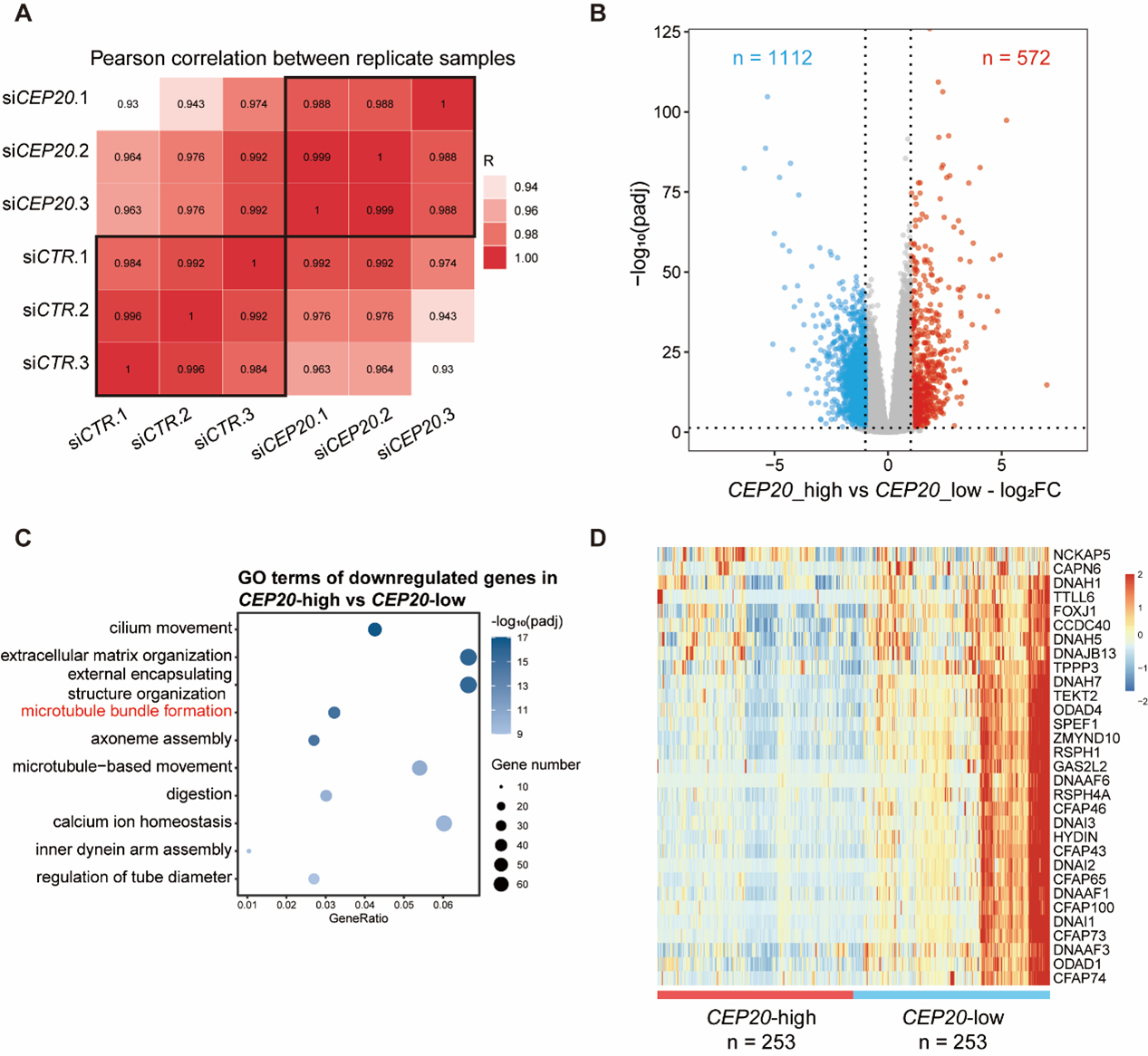

Supplement: Supplementary file 6 — Supplementary Information 6. [file 41598_2023_44754_MOESM6_ESM.tif]

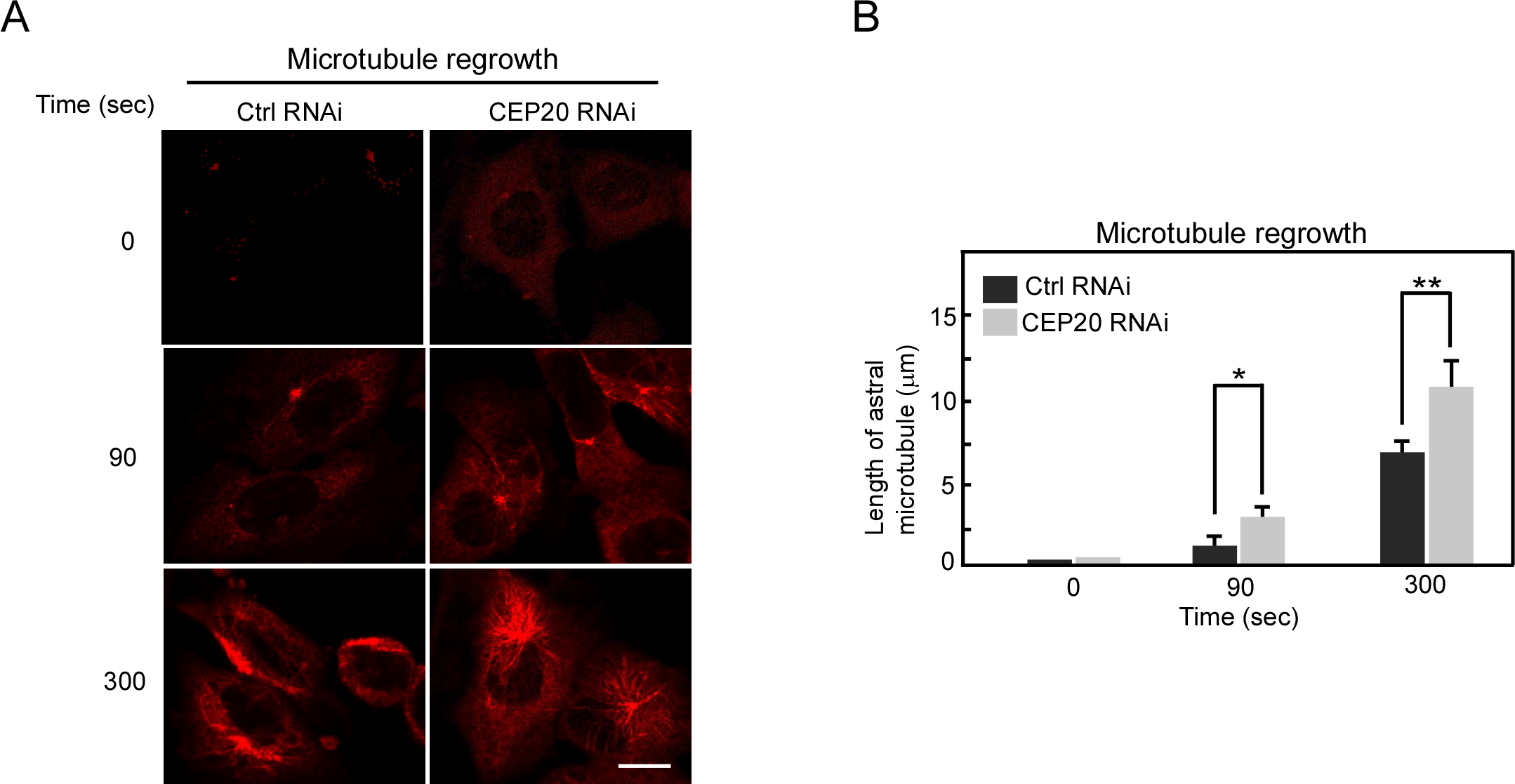

Supplement: Supplementary file 7 — Supplementary Information 7. [file 41598_2023_44754_MOESM7_ESM.tif]
