# Supplementary material for: CEP20 promotes invasion and metastasis of non-small cell lung cancer cells by depolymerizing microtubules
Source: Sci Rep. 2023 Oct 14;13:17484. doi: 10.1038/s41598-023-44754-8 (PMC10576744; doi:10.1038/s41598-023-44754-8)
Supplement: Supplementary file 9 — Supplementary Information 9. [file 41598_2023_44754_MOESM9_ESM.docx]

**Figure S1. CEP20 expression was higher in NSCLC tumor samples than adjacent normal samples in the TCGA and GEO databases.** (A) The expression of *CEP20* in 30 paired NSCLC tumor samples and adjacent normal samples from GSE19804. P = 0.0231, two-tailed Student’s t-test. (B) The expression of *CEP20* in 107 paired NSCLC tumor samples and adjacent normal samples from TCGA database. P = 0.0137, two-tailed Student’s t-test. (C) Violin and box plot showing the *CEP20* expression in unpaired NSCLC tumor samples and adjacent normal samples from TCGA database (tumor samples: n = 1035; normal samples: n = 108). P = 2.6e-20, two-tailed Wilcoxon test.

**Figure S2. Downregulation of endogenous CEP20 inhibits NSCLC cell proliferation in H1299.** (A) The protein expression of CEP20 by western blotting in H1299 cell. Actin was used as internal reference. Uncropped immunoblots were shown in Supplementary Fig. S8G. (B) The MTT cell viability assays were depicted in H1299 cell transfected with ctrl RNAi, CEP20 RNAi-1 or CEP20 RNAi-2, respectively. (C, D) The colony formation assays were performed in H1299 cell transfected with specific ctrl RNAi, CEP20 RNAi-1 or CEP20 RNAi-2, respectively. Bars indicate the mean ± s.d of three independent replicates. ** p < 0.01, *** p < 0.001.

**Figure S3.** **Downregulation of endogenous CEP20 inhibits NSCLC cell migration and invasion.** (A) The wound-healing assays showed the migration of the ctrl RNAi or specific CEP20 RNAi-1 or CEP20 RNAi-2. The dashed lines indicate the wound edges. Scale bar, 100 μm. The distance of the wound was measured by ImageJ software, and the data are presented as the mean ± standard deviation. (C) Transwell assays showed the migration of the ctrl RNAi or specific CEP20 RNAi-1 or CEP20 RNAi-1 and dyed with crystal violet staining. Scale bar, 50 μm. The quantitative data of the randomly selected fields are expressed as the mean ± standard deviation.

**Figure S4. Downregulation of endogenous CEP20 inhibits squamous cell carcinomas cell proliferation, cell migration and invasion in H226.** (A) The protein expression of CEP20 by western blotting in H226 cell. GAPDH was used as internal reference. Uncropped immunoblots were shown in Supplementary Fig. S8H. (B, C) The colony formation assays were performed in H226 cell transfected with specific ctrl RNAi, CEP20 RNAi-1 or CEP20 RNAi-2, respectively. (D) The wound-healing assays showed the migration of the ctrl RNAi or specific CEP20 RNAi-1 or CEP20 RNAi-2. The dashed lines indicate the wound edges. Scale bar, 100 μm. (E, F) Transwell assays showed the invasion of the ctrl RNAi or specific CEP20 RNAi-1 or CEP20 RNAi-2 and dyed with crystal violet staining. Scale bar, 50 μm. The quantitative data of the randomly selected fields are expressed as the mean ± standard deviation. *** p < 0.001.

**Figure S5. Downregulation of endogenous CEP20 inhibits squamous cell carcinomas cell proliferation, cell migration and invasion in H520.** (A) The protein expression of CEP20 by western blotting in H520 cell. Actin was used as internal reference. Uncropped immunoblots were shown in Supplementary Fig. S8I. (B, C) The colony formation assays were performed in H520 cell transfected with specific ctrl RNAi, CEP20 RNAi-1 or CEP20 RNAi-2, respectively. (D) The wound-healing assays showed the migration of the ctrl RNAi or specific CEP20 RNAi-1 or CEP20 RNAi-2. The dashed lines indicate the wound edges. Scale bar, 100 μm. (E, F) Transwell assays showed the invasion of the ctrl RNAi or specific CEP20 RNAi-1 or CEP20 RNAi-1 and dyed with crystal violet staining. Scale bar, 50 μm. The quantitative data of the randomly selected fields are expressed as the mean ± standard deviation. *** p < 0.001.

**Figure S6. The microtubule bundle formation related genes were highly expressed in CEP20-low NSCLC tumor samples.** (A) Spearman correlations between replicated RNA-seq samples (heatmaps of correlation matrix). (B) Volcano plot showing the differentially expressed genes in *CEP20*_high vs *CEP20*_low tumor samples from TCGA database. Red dots: upregulated genes in *CEP20*_high samples, blue dots: downregulated genes in *CEP20*_high samples. (C) Bubble plots of GO terms enriched in downregulated genes in in *CEP20*_high samples. Point sizes represent the gene numbers in each term, blue gradient represents the -log_10_ (adjusted P values). (D) Expression heatmap of microtubule bundle formation related genes in *CEP20*_high and *CEP20*_low tumor samples from TCGA database.

**Figure S7.** **Depletion of CEP20 changes the status of microtubule polymerization in A549 cells**. (A) A549 cells treated with nocodazole (5 μM) for 3 h and were washed out to allow microtubule regrowth for the indicated times and processed for immunofluorescence assay with anti-α-tubulin antibody. (B) The astral length of microtubules were evaluated by ImageJ software. Bar, 10 μm. Quantitative data of microtubule intensities are presented as mean ± SD. **P* < 0.05 and ***P* < 0.01, student’s *t*-test.

**Figure S8. Uncropped immunoblot images.** (A) The protein expression of CEP20 by western blotting in non-small-cell lung cancer tissues compared to the matched adjacent normal tissues. Actin was used as the internal reference. Red boxes indicate bands that were cropped for representative images. (B) The protein expression of CEP20 by western blotting in A549 cell. Actin was used as internal reference. Red boxes indicate bands that were cropped for representative images. The blots were cutted prior to hybridisation with antibodies. (C) Western blotting revealed the expression level of endogenous CEP20 and ectopic GFP-CEP20 in A549 cells. GAPDH was used as an internal control. Red boxes indicate bands that were cropped for representative images. The blots were cutted prior to hybridisation with antibodies. (D) Lysates from A549 cells transfected with the indicated plasmids were subjected to ultracentrifugation. The supernatant (S) and pellet (P) fractions were then processed for Western blotting with anti-α-tubulin and CEP20 antibodies. Red boxes indicate bands that were cropped for representative images. (E) Lysates from A549 cells transfected with the indicated plasmids and treated with nocodazole for the indicated times were subjected to ultracentrifugation. The supernatant(S) and pellet (P) fractions were collected separately and analyzed by western blotting with anti α-tubulin. Red boxes indicate bands that were cropped for representative images. (F) Lysates from A549 cells transfected with the indicated plasmids and treated with nocodazole for 3h to depolymerize microtubules, and then carefully washed out to remove nocodazole followed by fixation at the indicated times were subjected to ultracentrifugation. The supernatant(S) and pellet (P) fractions were collected separately and analyzed by western blotting with anti α-tubulin. Red boxes indicate bands that were cropped for representative images. (G) The protein expression of CEP20 by western blotting in H1299 cell. Actin was used as internal reference. Red boxes indicate bands that were cropped for representative images. The blots were cutted prior to hybridisation with antibodies. (H) The protein expression of CEP20 by western blotting in H226 cell. GAPDH was used as internal reference. Red boxes indicate bands that were cropped for representative images. (I) The protein expression of CEP20 by western blotting in H520 cell. Actin was used as internal reference. Red boxes indicate bands that were cropped for representative images.
